# Environmental surveillance of non-polio enteroviruses in Iran

**DOI:** 10.1186/1743-422X-6-149

**Published:** 2009-09-25

**Authors:** Mohammad Kargar, Sara Sadeghipour, Rakhshandeh Nategh

**Affiliations:** 1 Department of Microbiology, Islamic Azad University, Jahrom Branch, Iran; 2 Department of Virology, School of Public Health, Tehran University of Medical Sciences, Iran

## Abstract

**Background:**

Enteroviruses can shed in feces for several weeks, so many excrete viruses can remain infectious for a long time in environment. Therefore, by detecting enteroviruses in environmental specimens and sewage, we can understand this virus circulation, the approximate ratio of contaminated persons in society and they are suitable indicators for environmental surveillance.

**Methods:**

Since March 2006 to February 2007, 86 specimens from Sistan & Balouchestan,63 specimens from Tehran and 48 samples from Fars sewage disposal systems and surface water were collected by Grab Sample method and tested for enteroviruses directly by using two concentration methods: Pellet and Two-phase. Then Non-Polio Enteroviruses (NPEV) were serotyped by microneutralization method.

**Results:**

Enteroviruses were isolated from 49(56.98%) of specimens in Sistan & Baluchestan,38(60.32%) in Tehran and 11(22.92%) in Fars. Besides, the majority of Non-Polio Enteroviruses related to Non-typable Enteroviruses (N.T.E.V), E11 (31.52%), COX-B (27.58%), E7 (17.73%) and E4 (21.67%).

**Conclusion:**

Environmental surveillance has been used successfully in monitoring enteric virus circulation and assessing the extent or duration of epidemic non polioviruses in specific populations. The results of this research show the seasonal circulation of enteroviruses in different parts of Iran.

## Background

Enteroviruses were originally classified into four groups, polioviruses, coxsackie A viruses (CA), coxsackie B viruses (CB), and echoviruses, but it was quickly realized that there were significant overlaps in the biological properties of viruses in the different groups. The more recently isolated enteroviruses have been named with a system of consecutive numbers: EV68, EV69, EV70, and EV71 [1].

Human enteroviruses (family *Picornaviridae*) infect millions of people worldwide each year, resulting in a wide range of clinical outcomes ranging from unapparent infection to mild respiratory illness (common cold), hand, foot and mouth disease, acute hemorrhagic conjunctivitis, aseptic meningitis, myocarditis, severe neonatal sepsis-like disease, and acute flaccid paralysis. In the United States, enteroviruses are responsible for 30,000 to 50,000 meningitis hospitalizations per year as a result of 30 million to 50 million infections. Other types are coxsackie and echovirus. Enteroviruses are the most common cause of aseptic meningitis and can cause serious diseases especially in infants and the immunocompromised [2,3]. Transmissions of these viruses are usually by the fecal-oral or by the respiratory route [4]. Enteroviruses infection typically occurs in outbreaks during the tropical rainy seasons, or the temperate summer and autumn, mainly affecting young children. The risk of infection is directly correlated with poor hygiene and poor sanitation and overcrowding, typically among inadequately vaccinated populations [5]. To help public health officials recognize and control outbreaks of enteroviral disease, the National Enterovirus Surveillance System (NESS) is a voluntary, passive surveillance system that has monitored trends in circulating enteroviruses since 1961 in the United States. During 1970-2005, a total of 52,812 enterovirus detections were reported to NESS (29,772 of them during 1983-2005). Laboratory participation and the numbers of reports declined throughout the 1990s, but they increased again after 2000. The 15 most commonly reported enteroviruses accounted for 83.5% of reports with known serotype, and the five most commonly reported serotypes (echoviruses [E] 9, 11, 30, and 6, and coxsackievirus B5) accounted for 48.1%. Long-term circulation patterns for individual serotypes varied but were consistent with epidemic (e.g., E9, E13, E30, and coxsackievirus B5) or endemic patterns (e.g., coxsackieviruses A9, B2, B4, and enterovirus 71). Enterovirus detections had prominent summer-fall seasonality, with June-October accounting for 77.9% of reports with known month of specimen collection [3]. Fortunately, these virus isolation procedures detect non-polio enteroviruses (NPEV), either because these are the etiology of flaccid paresis in some cases or, if not related to flaccid paralysis, because they are shed with faeces as innocent bystanders. NPEV are endemic worldwide and multiple infections with various of the more than 70 types are usual. Precise information on the epidemiology of NPEV is fundamental for understanding the association of NPEV with serious diseases [6].

Virtually all countries adopted the four principal strategies for eradication, namely high routine immunization coverage, national immunization days (NIDs), a surveillance system for acute flaccid paralysis (AFP) with laboratory investigation, and mopping-up immunization activities [7]. Implementation of WHO-recommended strategies for poliomyelitis eradication resulted in a decrease in the number of globally reported poliomyelitis cases [8] and the number of countries in which poliovirus is endemic declined from 125 to 6 (Afganestan, Pakistan, Nigeria, Egypt, Niger, India) by 2003 [9-11].

During 2002-2004, a total of 24 laboratories, including 22 public health laboratories, one private laboratory, and the CDC Enterovirus Laboratory, reported 4,123 enterovirus detections in 46 states and Puerto Rico. The two predominant enteroviruses, echoviruses 9 and 30, accounted for more than half of all enterovirus detections in the United States during 2002-2004. Echovirus 9 accounted for 21.5%,41.0%, and 18.9% of detections with known serotypes during 2002, 2003, and 2004, respectively. Echovirus 30 was uncommon in 2002 (3.3%) but accounted for 32.4% of reports with known serotypes in 2003 and 40.3% in 2004. During this period, echovirus 9 was detected in 41 states and Puerto Rico, echovirus 30 in 38 states and Puerto Rico, and echovirus 7 in 24 states. Three states of USA (Georgia, Illinois, and New York) accounted for 528 (47.8%) of the echovirus 9 detections [3].

Therefore, WHO has suggested environmental surveillance using surface water and sewage specimens in high risk rigions [10,11].

The aim of this study was environmental surveillance by using sewage and surface water to evaluate environmental and seasonal circulation of non polio enterovirus (NPEV) in three main provinces of Iran.

## Materials and methods

### Sampling

In this study, since March 2006 to February 2007, 86 samples from 2 sewage disposal systems, 5 hospitals and surface water from several villages in Sistan-Balouchestan,63 samples from 6 sewage disposal systems in Tehran and 48 samples from 2 Hospitals and surface water in Fars Province were collected using Grab Sampling procedure. All the samples were collected from the influent of raw sewage. Samples were collected in 1000 ml sterile bacteriological sampling bottles and were carried to National Polio Laboratory in Tehran University of Medical Science Research Institute. In all cases, the characteristics of sewage samples (place, date, pH, and temperature) were documented. The samples during transferring and before inoculation to cell culture, kept at 4°C (cold chain).

### Concentration

The sewage samples were examined directly and also by two concentration methods: Pellet and Two-phase. It is worthy to say that, the Pellet method, for the first time, is suggested by us. To concentrate by this method the supernatant was transferred to a sterile flask. Then from the remainder of sewage, 75 ml was transferred to 5 sterile centrifuge tubes and it was centrifuged for 10 min with 5000 rmp at 5°C and the tubes were kept at 4°C. The Two-phase method was accomplished by using the suggested method of Hovi in 2001 [12]. For destroying the bacteria and fungus 1 ml of chloroform were added to 4 ml of the Direct, Pellet and Two-phase samples and were shake for 20 min whit 200 rpm. The containers of the tubes were centrifuged in 2000 rpm at 5°C and supernatant was collected in 1.8 ml sterile cryotube.

### Cell culture method

For isolation of non-polio enteroviruses (NPEVs) the RD and HEp-2 cell lines are used. The sewage inoculation rate to each tube of cell culture was 200 μl. After inoculation they were kept in 36°C for 7 d. To observe the CPE, the tubes were examined by inverted microscope every day and the positive samples were kept at -20°C. Also after 7 d, the negative tubes were Freezed & Thawed and re-passaged in RD and HEp-2[13].

### Neutralization test

For the identification of non polio enteroviruse isolates, samples of diluted isolate were mixed with equal volumes of a selected set of polyclonal antisera made in animals against a trivalent pooled polio antiserum (PP), a coxsackievirus B1-B6 pool (CP), and seven pools against coxsackievirus A9 and 20 echoviruses (A-G). Using the micro-neutralization technique, the antisera-virus mixtures were incubated for 1 h at 36°C to allow the antibodies to bind to the virus. Subsequently, suspensions of cells were added to the microtitre plate which were examined daily for the presence of CPE. The antiserum that prevented the development of CPE indicated the identity of virus [13].

### Statistical analysis

The data were described using analytical statistics. A value of P < 0.05 was considered statistically significant. We used *SPSS *Ver 13 for analysis data.

## Results

**Figure 1:**
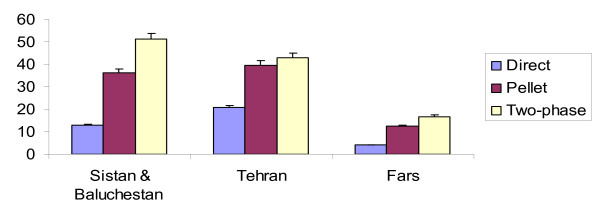
**Number of isolated Non-polio Enteroviruses based on three concentration methods in Sistan and Balouchestan, Tehran and Fars**.

**Figure 2:**
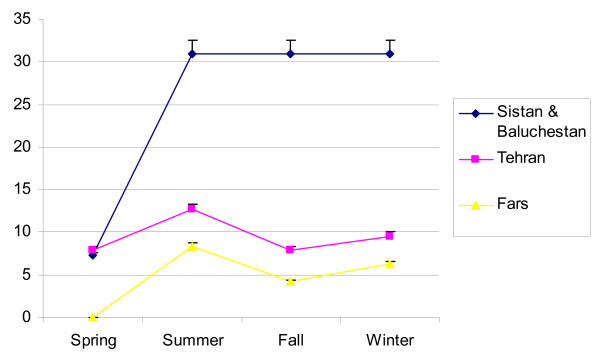
**Number of Non-polio Enteroviruses based on different seasons in Sistan & Baluchestan, Tehran and Fars**.

Eighty six samples from two sewage disposal systems, 5 hospitals and number of villages in Zabol, Zahedan and Chabahar cities,63 samples from Tehran and 48 samples from Fars Provinces were collected. From the 86 collected samples in Sistan & Balouchestan Province the most isolated NPEV related to E4, COX-B, E11, Non-typable Enteroviruses (NTEV) and E7 with 20,16.36,14.55,12.73 and 10.91 percent, respectively. Out of 63 samples in Tehran the most isolated NPEV serotypes serotypes regarded to NETV, E11, E25, E20 with 22.58,12.90, 12.90,9.68 and 9.68 percent and from forty eight samples in Fars, the most isolated related to 11(44.44%),,NETV(22.22%),,COX-B(22.22%) and E7 (11.11%), respectively (Table 1). The isolation of NPEV in Sistan & Balouchestan by Direct, Pellet and Two-phase concentration methods were 11(12.79%),31(36.05%)and 44(51.16%)respectively (Fig. 1). Statistical analysis with SPSS13 software were reflected that there was no significant correlation between Direct method and Pellet & Two-phase concentration methods for detection of NPEV in Sistan & Baluchestan Province. This matter indicates the acceptability of Pellet and Two-phase methods for isolation of NPEV. But there was significant correlation (in 0.01 level) between Direct method and Pellet & Two-phase concentration methods in Fars and Tehran Provinces. According to the Fig. 2 Sistan & Baluchestan has the greatest number of isolated N.P.E.V, as well as, the isolation in the summer, autumn and winter were the same (30.91%) and the lowest circulation related to spring (7.27%). As the graph shows, the isolation of N.P.E.V in spring and autumn were the same in Tehran Province. Meanwhile the isolation in summer and winter revealed the same pattern. Besides, the most isolation of NPEV in Fars regarded to summer (4.16%), winter (6.25%) and autumn(4.16%). As a whole, there was no significant correlation between isolation of Enteroviruses and different seasons. Moreover the isolation of NPEV in RD and HEp-2 cell lines indicate that, RD cell line is the best for detection of NPEV in Sistan & Balouchestan Province with 53.94% and also the detection was 10.47% in HEp-2 cell line. Having applied *SPSS 13 *and *ANOVA test*, there was significant correlation for isolation of NPEV between RD and HEp-2 cell lines. In Fars the best cell line for isolation of NPEV was RD cell line with 16.6% and the isolation in HEp-2 cell line was 4.17%, all of isolated virus in HEp-2 related to COX-B virus, and the isolation of NPEV in Tehran were 44.66% in RD and 15.87% in HEp-2 cell line too.

**Table 1:** Number of isolated Non-polio Enteroviruses in this study, Iran, Sistan and Balouchestan, Tehran and Fars

**Serotype**	**N.T.E.V**	**E1**	**E3**	**E4**	**E6**	**E7**	**E11**	**E12**	**E13**	**E20**	**E21**	**E25**	**E27**	**E33**	**COX-B**	**Total**
**Provinces**	**n(%)**															

**Sistan & Baluchestan**	7(12.73)	2(3.64)	3(5.45)	11(20)	4(7.27)	6(10.91)	8(14.55)	4(7.27)	0(0)	0(0)	0(0)	0(0)	0(0)	1(1.82)	9(16.36)	55(100)
**Tehran**	7(22.58)	1(3.22)	0(0)	0(0)	1(3.22)	2(6.45)	4(12.9)	0(0)	3(9.68)	3(9.68)	1(3.22)	4(12.9)	2(6.45)	0(0)	3(9.68)	31(100)
**Fars**	2(22.22)	0(0)	0(0)	0(0)	0(0)	1(11.11)	4(44.44)	0(0)	0(0)	0(0)	0(0)	0(0)	0(0)	0(0)	2(22.22)	9(100)
**Total**	16(31.52)	3(5..91)	3(5..91)	11(21.67)	1(1..97)	9(17.73)	16(31.52)	4(7.88)	3(5..91)	3(5..91)	1(1.97)	4(7.88)	2(3.94)	1(1.97)	14(27.58)	95(100)

## Discussion

Monitoring circulating enteroviruses is important because individual serotypes have different temporal patterns of circulation and the changes in predominant serotypes can be accompanied by large-scale outbreaks of enteroviral illnesses. Serotype-based enterovirus surveillance in the United States has five objectives. First, NESS data help public health practitioners determine long-term patterns of circulation for individual enteroviruses. Moreover, the data are used for interpreting trends in enteroviral diseases, such as aseptic meningitis, by associating them with circulating serotypes and can be helpful for studying the association of enteroviruses with clinical manifestations. Besides, the data are used to guide outbreak investigations by enabling linkage of disease clusters; diagnosis by serologic assay and clinical presentation, which varies by serotype; and timelier laboratory identification. Likewise, because susceptibility to candidate anti-enterovirus drugs varies by serotype, information on circulating serotypes helps guide development of new diagnostic tests and therapies. Finally, NESS monitors poliovirus detections, thereby supplementing poliovirus surveillance in the United States [3]. However, NPEV are occasionally related to more serious illnesses, for example aseptic meningitis, life-threatening myocarditis and hepatitis, and are probably associated with juvenile diabetes mellitus type 1. Compared to the number of NPEV infections, these serious organ infections are rather rare events, with a frequency quite similar to that of poliomyelitis anterior, which is an infrequent organ manifestation of poliovirus wild-type infection, and an extremely rare complication of poliovirus vaccine strains. Nevertheless, such a strategy is justified if the study investigates the association of NPEV types with a certain disease [6].

In the context of poliomyelitis eradication, a reinforced sentinel laboratory network for surveillance of enteroviruses (RSE) was implemented in France in January 2000, and the purpose of that report is to describe the results of the five first years of surveillance. Over the 5 years of surveillance, information was collected from 192,598 clinical samples, including 39,276 cerebrospinal fluid specimens, of which 14.7% were positive for enteroviruses, 45,889 stool samples (4.3% positive for enteroviruses), 70,330 throat swabs (2.2% positive) and 14,243 sera (1.4% positive). The ten main non-polio enteroviruses typed were as follows, in decreasing order of frequency: E-30, E-13, E-6, CV-B5, E-11, CV-B4, E-9, E-7, CV-B1, and CV-B2. Continued surveillance of enteroviruses is important to alert physicians and public health officials to changes in disease trends. Although the geographical coverage of the RSE network as well as the percentage of enteroviruses identified must be improved, the large number of samples tested for enteroviruses shows the ability of virology laboratories to detect the circulation of enteroviruses and to report the possible identification of poliovirus (wild-type, vaccine-derived, or Sabin-like) [14]. In several countries wild polioviruses have been detected in the environment in the absence of reported AFP cases. Thus, after eradication of wild polioviruses from AFP cases in high risk areas, WHO has recommended the complementary surveillance by using sewage sample and stools of healthy children [11]. Therefore, Sistan & Balouchestan, Tehran and Fars provinces were selected for this research. Based on the recommendation of WHO, a useful criterion of satisfactory overall performance of the surveillance is detection of non-polio Enteroviruses in the samples. At least 30% of concentrated sewage from grab samples should reveal NPEV [10,11].

In this study, for the first time, we suggested the Pellet concentration method, and used the Two-phase concentration method, simultaneously. From the total samples in Sistan & Baluchestan, non-polio enteroviruses were isolated from 11(12.79%), 31(36.05%) and 44 (51.16%) samples by direct, pellet and two-phase methods, respectively. These results confirm the efficiency of concentration methods, in enterovirus surveillance. Another purpose of this study was evaluation of distribution and analysis of environmental circulation of NPEVs. Japanes, study on Enteroviruses shows that E6, E17, Cox-B5 in 1999, E9, E71, E25, E11 in 2000 and E11 and Cox-B5 in 2001 have played the main role in aseptic meningitis outbreak. In 2002, also E11 and E13 were the most frequently isolated Enteroviruses from aseptic meningitis patients [15]. During the seasons under study, E4 (20%), Cox-B (16.36%) and E11 (14.55%) were the predominant serotypes in Sistan & Baluchestan. But N.T.E.V(22.58%), E25 and E11(12.9%) were the most serotypes in Tehran Province. The epidemiological pattern of enterovirus infections varies by geographical region, climate, age and season. Therefore, it is necessary to evaluate relationship between non-polio enterovirus disease and environmental circulation of these viruses in different part of Iran. Such studies can be perform for providing a suitable vaccine to prevent of enterovirus infections in high risk area. Until now, the cell line that capable to isolation of all enteroviruses has not identified. Several coxsackievirus A (CAV) serotypes of the species *Human enterovirus A *are hard to isolate on cell cultures and require animal experiments with suckling mice for virus isolation. These are not routinely performed in most laboratories. Fortunately, RD cells are recommended by the World Health Organization for poliovirus surveillance. Use of RD cells and of the shell vial technique clearly improves isolation of CAV serotypes but some serotypes and strains even fail to replicate on RD cells. Thus poliovirus surveillance efforts may produce some data on CAV circulation but some CAV types are still overlooked by this approach, leaving the picture of enterovirus surveillance somewhat incomplete [6]. However, the use of L20B and RD cells without HEp-2, may have an impact on the non-poliovirus enterovirus isolation rate, especially during periods of Coxsackie B circulation in the community [13,16,17]. Therefore, in this study RD and HEp-2 cells were used for identification of more extend spectrum of enteroviruses. Overall, 46 and 9 NPEVs were detected in Sistan & Baluchestan, 28 and 10 NPEVs in Tehran, 7 and 2 NPEVs in Fars, on RD and HEp-2 cells, respectively [9,18]. Not isolating vaccine derived polioviruses (VDPV) and vaccine derived NPEV shows the proper AFP surveillance and vaccination coverage in our country at high risk areas. But, repeated sampling and environmental surveillance will increase the probability of detecting low level transmission of enteroviruses in population.

## Competing interests

The authors declare that they have no competing interests.

## Authors' contributions

MK carried out the design of the study, coordination and performed the statistical analysis. SS participated in sampling, concentration, cell culture and neutralization test. RN participated in the scientific consultation of this research project. All authors read and approved the final manuscript.
